# Survival analysis of spontaneous speech for predicting mild cognitive impairment-to-dementia conversion

**DOI:** 10.1016/j.tjpad.2026.100692

**Published:** 2026-11-03

**Authors:** Adrián Hinojosa-Calleja, Berta Calm, Montserrat Alegret, Ana Espinosa, Amanda Cano, Maria Victoria Fernández, Andrea Miguel, Ariadna Solivar, Alejandro Valenzuela-Seba, Pablo García-González, Raquel Puerta, Clàudia Olivé, Maria Capdevila-Bayo, Álvaro Muñoz-Morales, Paula Bayón-Buján, Marta Rovira-Bruna, Maider Iza, Laura Montreal, Maitee Rosende-Roca, Pilar Sanz-Cartagena, Yahveth Cantero-Fortiz, Miren Jone Gurruchaga, Lluis Tarraga, Xavier Montalbán, Mercè Boada, Agustín Ruiz, Marta Marquié, Sergi Valero

**Affiliations:** a Ace Alzheimer Center Barcelona - Universitat Internacional de Catalunya, Gran Via Carles III 85 bis, Barcelona, 08028, Spain; b Networking Research Center on Neurodegenerative Diseases (CIBERNED), Instituto de Salud Carlos III, Av. Monforte de Lemos, 3-5, Madrid, 28029, Spain; c PhD Program in Biotechnology, Faculty of Pharmacy and Food Sciences, University of Barcelona, Av. De Joan XXIII, 27-31, Barcelona, 08028, Spain; d Department of Pharmacology, Toxicology and Therapeutic Chemistry, Faculty of Pharmacy and Food Sciences, University of Barcelona, Av. De Joan XXIII, 27-31, Barcelona, 08028, Spain; e Universitat de Vic-Universitat Central de Catalunya (UVic/UCC), C. Sagrada Familia 7, Vic, 85000, Spain; f Glenn Biggs Institute for Alzheimer’s & Neurodegenerative Diseases, University of Texas Health Science Center, 8300 Floyd Curl Drive, San Antonio, TX, 78229, USA; g Department of Microbiology, Immunology and Molecular Genetics, Long School of Medicine, University of Texas Health Science Center, 8300 Floyd Curl Drive, San Antonio, TX, 78229, USA

**Keywords:** Alzheimer’s disease, Mild cognitive impairment, Speech biomarkers, Dementia risk, Survival analysis

## Abstract

**Background:**

Speech is emerging as a promising digital biomarker of brain health. However, most speech-based studies in MCI and dementia have focused on cross-sectional diagnostic classification rather than longitudinal prognosis. Although speech-derived features have recently been used to predict MCI-to-dementia progression within fixed follow-up windows, their value for estimating time-to-dementia conversion within a survival-analysis framework remains insufficiently characterized.

**Objectives:**

To assess whether spontaneous speech-derived features serve as prognostic digital biomarkers for time-to-dementia conversion in individuals with MCI, and to benchmark their performance against established cognitive composite measures.

**Methods:**

We conducted a longitudinal prognostic study in patients with MCI evaluated at the Ace Alzheimer Center Barcelona Memory Clinic from 2022 onward. At baseline, participants completed a brief spontaneous speech protocol from which multi-domain linguistic and acoustic features were automatically extracted. Patients underwent standard annual clinical follow-up to determine conversion to dementia. Baseline speech features were used to train survival models estimating conversion risk for all participants, and performance was evaluated using the C-index, 2-year time-dependent AUC, and IBS. Predictive accuracy was benchmarked against models trained on cognitive composite scores.

**Results:**

A total of 853 individuals diagnosed with MCI were included at baseline, of whom 187 converted to dementia during follow-up. Cognitive composite models showed the strongest prognostic performance, achieving a test-set C-index of 0.77, 2-year horizon AUC of 0.82, and IBS of 0.12. Among speech-derived feature sets, Semantic Verbal Fluency showed the best performance, with a test-set C-index of 0.74, 2-year AUC of 0.74, and IBS of 0.12.

**Conclusion:**

Semantic Verbal Fluency-derived speech features demonstrated good prognostic performance for MCI-to-dementia conversion, although cognitive composites remained the strongest predictors. These findings suggest that a brief, automatically processed verbal-fluency task may provide a scalable and low- burden complement to standard cognitive assessment for dementia risk stratification, particularly in settings where repeated or remote monitoring is desirable. External validation and longer follow-up are needed before clinical implementation.

## 1 Introduction

Mild cognitive impairment **(MCI)** is characterized by a measurable decline in memory and other cognitive functions that exceed normal age-related changes but does not compromise an individual’s ability to carry out everyday activities independently. It represents an intermediate clinical stage between cognitively healthy aging and dementia.

Alzheimer’s disease **(AD)** is the leading cause of dementia worldwide, accounting for 60–70% of cases. In general, MCI progression to AD occurs at an annual rate of 10–25% [1]. It was estimated that in 2019, 55.2 million people worldwide were living with dementia [2], and this number is projected to reach 78 million people in 2030 and about 139 million in 2050 [3]. Despite this growing burden, the ability to identify individuals at high risk of progression during the prodromal stage remains limited. This gap has driven increasing interest in scalable, low-burden biomarkers capable of capturing early disease dynamics beyond conventional clinical assessments.

Recently, automated speech analysis has emerged as a particularly promising approach for the early detection of dementia [[4], [5], [6], [7]]. Language impairments often manifest years before progression to the dementia stage, offering valuable insights into MCI [8,9]. Speech-based diagnostic approaches leverage advanced Machine Learning **(ML)** and Natural Language Processing **(NLP)** techniques to analyze linguistic and paralinguistic features extracted from spontaneous speech [10]. This approach not only enables more cost-effective, non-invasive, and remote longitudinal assessments, but also aligns with the growing trend toward e-health technologies and decentralized clinical trials [11].

Survival analysis **(SA)**-based ML models have recently emerged to predict the risk of conversion from MCI to dementia. Notably, most of them rely on conventional diagnostic modalities as predictors, such as neuropsychological assessments [12,13]. Many models further rely on cerebrospinal fluid biomarkers, magnetic resonance imaging, or positron emission tomography measures [[14], [15], [16], [17], [18], [19], [20], [21], [22]].

In this context, the present study aims to determine whether speech-derived features can serve as predictors of conversion from MCI to dementia within a SA framework. To contextualize the incremental/prognostic value of speech-derived features, two additional predictor categories were considered: demographic variables and cognitive composite measures. Separate survival models were trained for each feature category to enable a structured comparison of their prognostic performance. This design allowed us to assess the relative contribution of speech-derived markers alongside established clinical predictors and to determine their potential utility for scalable risk stratification.

## 2 Methods

### 2.1 Study participants

This study included data from individuals diagnosed with MCI at the Memory Clinic of Ace Alzheimer Center Barcelona **(Ace)**, with baseline assessments conducted between March 2022 and April 2024. Diagnoses were determined by a multidisciplinary team comprising neurologists, neuropsychologists, and social workers during a consensus diagnostic conference.

The diagnosis of AD is based on the National Institute of Neurological and Communicative Disorders and Stroke–Alzheimer’s Disease and Related Disorders Association criteria. Standard diagnostic criteria were used for the classification of vascular dementia, frontotemporal dementia, Lewy body dementia, Parkinson’s dementia, and other neurodegenerative diseases [23]. Additionally, blood samples were collected from all participants for genetic analysis to determine the presence of the apolipoprotein E (**APOE**)-*ε*4 allele [24].

Following the baseline diagnosis, all patients underwent annual cognitive re-evaluations at Ace. During follow-up visits, a similar assessment sequence was used: the neuropsychological evaluation was repeated in full, whereas the neurological examination was more focused.

The most recent assessments were conducted in July 2025. The date of conversion was de- fined as the first visit at which a participant met criteria for dementia. For participants who did not convert, censoring time was defined as the date of the last available clinical assessment. MCI was defined as cognitive complaints with objective impairment on standardized tests adjusted for age and educational level, with preserved functional autonomy [25], corresponding to Clinical Dementia Rating (**CDR**) = 0.5. Dementia was defined as cognitive impairment interfering with functional independence corresponding to CDR > 0.5. Participants who remained at CDR = 0.5 during follow-up were classified as stable MCI **(sMCI)**, whereas those who progressed to CDR > 0.5 were classified as progressive MCI **(pMCI)**.

#### 2.1.1 Calculation of cognitive composites

Cognitive composite scores were derived from the Neuropsychological Battery of ACE **(NBACE)**, described in [26], and were used to determine participants’ cognitive status. Scores were calculated across seven cognitive domains: reality orientation, attention, memory, language, praxis, visuospatial/visuoperceptual abilities, and executive functions.

Composite scores were calculated using structural equation models **(SEM)** and defined by an expert panel of neuropsychologists. All composite scores were adjusted for age, sex, and years of formal education. A detailed description of the SEM methodology is provided elsewhere [6].

### 2.2 Recording protocol and speech feature extraction

The speech protocol was administered in Spanish using the AcceXible platform on a tablet within a quiet and controlled environment. The protocol included three one-minute tasks:

(1) image description **(IMG)**, in which participants were presented with *The Cookie Theft Picture* [27] and asked to provide a detailed description of the image; (2) semantic verbal fluency **(SVF)**, in which participants were asked to list as many animals as possible; (3) autobiographical recall **(PPE)**, in which participants were asked to recount a positive personal experience from the past month.

Audio recordings from the three tasks were transcribed using the *Whisper* large-v2 speech- to-text model [28]. The resulting transcriptions were subsequently processed using the *Stanza* NLP library to perform part-of-speech tagging and assign lexical categories to each word [29]. The audio recordings, their corresponding transcriptions, and the derived linguistic annotations constituted the primary database sources for feature extraction. From these materials, a multi-domain set of speech variables was computed, including acoustic, lexical, syntactic, semantic, and task-specific features. Acoustic variables characterized voice production and paralinguistic properties; lexical and syntactic variables quantified vocabulary usage and grammatical structure; semantic features captured conceptual and distributional aspects of discourse; and task-specific variables were designed to leverage the structural properties of each speech task. A detailed description of the processing procedures and feature computation methods are provided in Section 2.5 of [7].

### 2.3 Data preparation

The final dataset comprised 1345 features organized into the following categories:

• Demographic and *APOE* -*ε*4 **(DEA)**: age, sex, years of formal education and *APOE*
• *ε*4 allele carrier status.
• Cognitive composites **(COM)**: seven composite scores for reality orientation, attention, memory, language, praxis, visuospatial/visuoperception, and executive functions.
• Image **(IMG)**: 493 features extracted from the image description task.
• Semantic Verbal Fluency **(SVF)**: 434 features extracted from the semantic verbal fluency task.
• Positive Personal Experience **(PPE)**: 407 features extracted from the autobiographical recall task.

The sMCI/pMCI status and time to censoring or conversion to dementia in days were included as outcome variables for training the survival analysis models. Missing values were observed in the PPE and IMG categories, primarily due to instances where the participant spoke very little, limiting the extraction of certain features. To avoid data leakage, imputation was performed after splitting the data into training and test sets. For each feature category, the *MissForest* algorithm [30] was fitted only on the training data and then applied to both the training and test sets.

### 2.4 Model training and feature selection

In this study, we used SA models primarily to produce individualized risk scores (e.g., hazard-based rankings) that quantify each participant’s relative likelihood of earlier con- version compared with others. Specifically, we implemented the following ensemble survival models: Random Survival Forest **(RSF)**, Extra Survival Trees **(EST),** and Gradient Boosting **(GB)**.

Because censoring precludes the use of conventional machine-learning evaluation met- rics, predictive performance was assessed using Harrell’s concordance index **(C-index)** and the Integrated Brier Score **(IBS)** [31,32]. The C-index was used to evaluate discriminative ability, whereas the IBS was used to assess overall prediction error over time. In addition, we evaluated the ability of each model to identify short-term converters using the cumulative/dynamic area under the curve **(AUC-cd)** at a fixed two-year horizon, corresponding to 730 days after baseline [33].

Survival analysis was conducted using the Python package *Scikit-survival* [34]. The dataset was randomly split into training and test sets using a fixed random seed, with an 80:20 allocation, stratified by the conversion variable to preserve the original outcome distribution. Survival models were trained independently for each predefined feature category to assess their individual predictive contributions. In addition to these category-specific models, an additional survival model was trained using the combined feature set **I+S+P**, defined as the union of IMG, SVF, and PPE variables.

For each model and feature set, hyperparameters were first optimized using five-fold cross-validation **(CV)** on the training data, with the C-index used as the optimization metric. The best-performing hyperparameter configuration was then used in a Boruta-based feature selection procedure applied within a five-fold CV framework [35]. In each internal fold, the model was fitted on the augmented training partition containing both original and randomly permuted shadow features, and permutation importance was estimated on the corresponding validation partition using the survival C-index with 16 repetitions. A feature was counted as successful in a Boruta iteration when its mean permutation importance exceeded the maximum mean importance of the shadow features. Evidence across iterations was summarized with binomial tests and corrected for multiple comparisons. Features selected in at least three of the five internal folds were retained.

After feature selection, a second hyperparameter optimization step was performed, this time training the models only with the selected features. The resulting optimized models were subsequently fitted on the training set and evaluated on the independent test set to estimate their final predictive performance.

### 2.5 Analysis of speech features

Risk prediction scores were generated for all participants in the test set using the trained models. Participants with risk scores in the fourth quartile were classified as the high-risk group, whereas those with scores in the first quartile were classified as the low-risk group. Two-year survival probabilities for each group were estimated using the Kaplan–Meier (KM) method. In addition, for each extracted feature, mean values between the high-risk and low-risk groups were compared using a two-sample *t*-test to identify features that differed significantly between the groups.

## 3 Results

### 3.1 Characteristics of participants

A total of 853 individuals with MCI were included in this study, of whom 187 (21.9%) converted to dementia during follow-up. Mean observed follow-up was 1.68 years (SD 0.56) among participants who remained stable and 1.42 years (SD 0.55) among participants who converted to dementia. At the time of conversion, 154 patients were diagnosed with AD, 20 with vascular dementia, 5 with Lewy body dementia, 2 with Parkinson’s disease dementia, and 6 with other neurodegenerative diseases. Demographic and clinical characteristics are summarized in Table 1.

**Table 1:** Demographic and clinical characteristics of sMCI and pMCI groups.

Variable	sMCI	pMCI
Sample size	666	187
Age (mean, SD)	72.52 (8.90)	77.59 (5.79)
Sex (% Female)	61.56	69.52
Years of education (mean, SD)	8.62 (4.40)	7.48 (4.40)
MMSE (mean, SD)	27.05 (2.47)	24.62 (3.07)
*APOE ε*4 (%)	28.52	33.68
Marital status (%)		
* Married/Partnered	58.10	60.96
* Widowed	12.76	26.20
* Separated/Divorced	4.95	4.81
* Single	2.70	4.81
* Unknown	21.47	3.21
Cohabitation status (%)		
* Lives alone	13.96	29.94
* Lives with spouse/partner	54.50	57.75
* Other	9.30	9.10
* Unknown	22.22	3.21
Comorbidity (%)		
* Depression	37.30	27.87
* COPD	7.04	7.61
* Hypothyroidism	13.46	16.22
* Cardiopathy	8.41	9.09
* Arrhythmia	4.35	6.42
* Diabetes	14.44	19.89
* Hypertension	49.69	60.22
* Dyslipidemia	50.53	52.15
* Ischemic stroke	4.42	4.32

### 3.2 Feature selection

Missing values were imputed for 9 features in the PPE category and 11 features in the IMG category, with percentages of missingness ranging from 0.1% to 6.6%. Because of their limited number of predictors, feature selection was omitted for the DEA and COM categories.

Feature selection did not retain any variables in the IMG or PPE categories; therefore, models for these categories were trained using the full feature sets. In contrast, feature selection identified relevant predictors in the SVF category. Specifically, 5 features were selected by the RSF model, 8 by the EST model, and 5 by the GB model. Among these, three features were consistently selected across all models: unique animal counts, log mean language-use of words excluding function words, and the standard deviation of the F1 bandwidth falling slope.

For the combined I+S+P category, only features belonging to the SVF domain were selected. Given that the selected I+S+P features were exclusively SVF-derived, the results for the I+S+P category were highly similar to those obtained for SVF alone. Consequently, we decided to omit the I+S+P results from the analyzes.

### 3.3 Models’ performance

The best-performing models for each category were identified based on the mean C-index obtained during CV and evaluated on the test set. For the COM feature set, GB performed the best with 0.772 a mean C-index of 0.772 in CV and 0.769 on the test set. For the SVF feature set, EST performed best with a mean C-index of 0.729 in CV and 0.740 on the test set. For DEA, EST performed best, with a mean C-index of 0.668 in CV and 0.650 on the test set. For IMG, GB performed best, with a mean C-index of 0.640 in CV and 0.578 on the test set. Overall, COM showed the strongest discrimination, while SVF provided the best discrimination among speech-derived feature sets.

When model performance was assessed using 2-year horizon AUC, GB again performed best for the COM feature set, with a mean AUC of 0.795 in CV and 0.824 on the test set. For SVF, RSF achieved the highest AUC, with 0.746 in CV and 0.739 on the test set. For DEA, GB obtained the best performance, with 0.696 in CV and 0.636 on the test set. For IMG, EST performed best, with 0.655 in CV and 0.693 on the test set. For PPE, GB achieved the highest AUC, with 0.585 in CV and 0.671 on the test set. These results indicate that COM and SVF were the most informative feature categories for identifying short-term converters at the 2-year horizon.

Using IBS as the evaluation metric, RSF performed best across all the categories. For the COM feature set, RSF achieved a mean IBS of 0.116 in CV and 0.115 on the test set. On SVF with 0.123 on CV and 0.121 on test. On DEA, with .131 CV and 0.135 test. On IMG, with 0.136 CV and 0.139 test. On PPE, with 0.139 CV and 0.132 test. The lower IBS values observed for COM and SVF suggest better overall prediction accuracy over time compared with the other feature categories. A comparison of C-index, AUC and IBS performance across all feature categories is shown in Fig. 1.

**Fig. 1:**
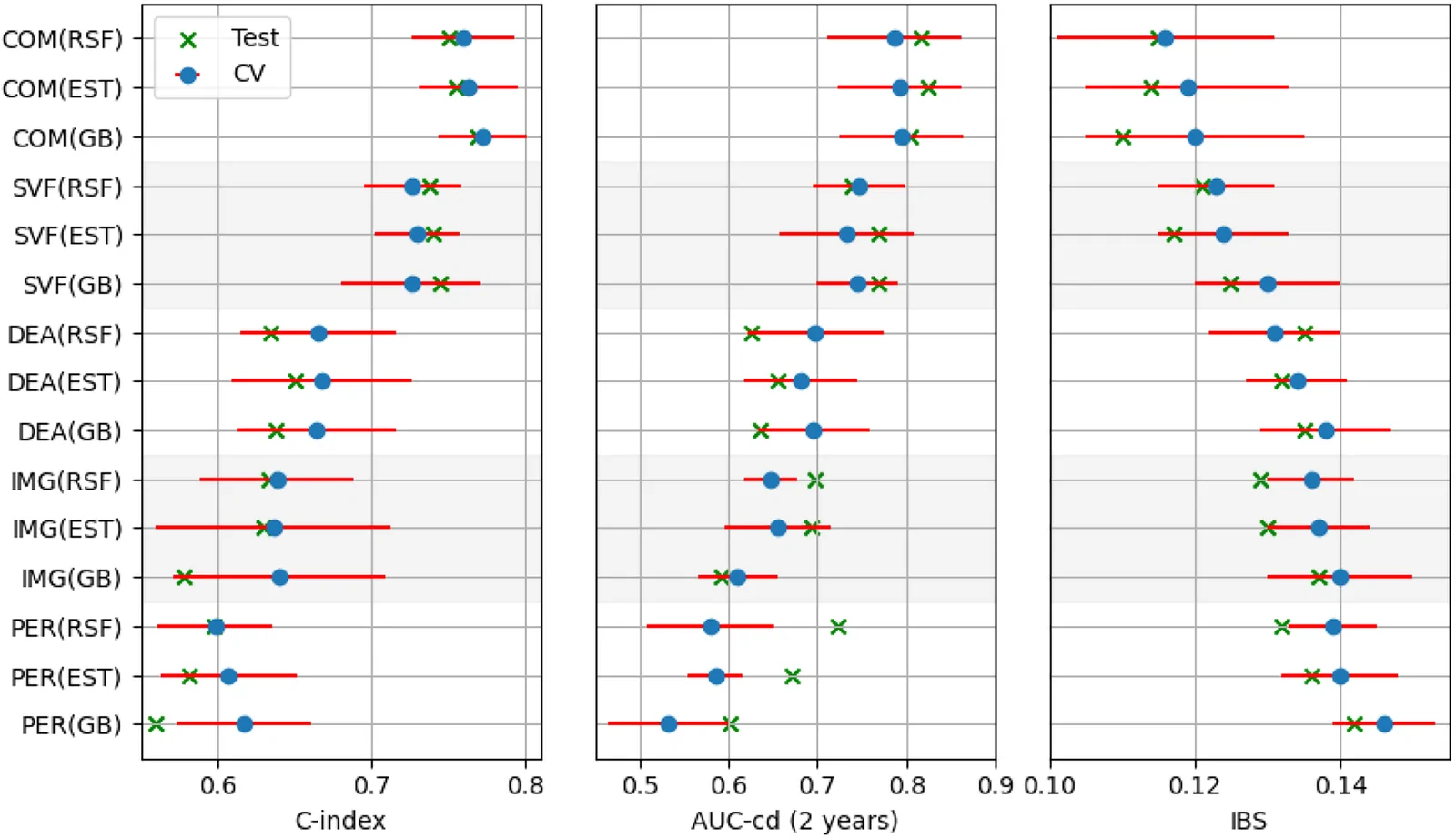
Comparison of model performance across feature categories and modeling approaches, C-index, time-dependent AUC at 2-year horizon, and IBS are shown in the left, middle, and right panels, respectively. Results are shown for the test set (green crosses) and CV (blue dots), with horizontal bars indicating SD.

### 3.4 Risk group analysis

Two-year KM estimated survival percentages for low and high risk across all feature categories are presented in Table 2. Complementary KM survival curves are plotted for COM, SVF, and DEA in Fig. 2. Regarding the two-sample *t*-test analyzes, several SVF selected features showed significant differences between the high-risk and low-risk groups. Their definitions and mean values by risk group are reported in Table 3.

**Table 2:** Two-year KM estimated survival percentages for low- and high-risk groups in the test set across feature categories and model types.

Model	Two-year KM % Survival (95% CI)	
	Low Risk	High Risk
COM (RSF)	97.56 (83.92–99.65)	56.29 (36.25–72.22)
COM (EST)	97.61 (84.27–99.66)	52.53 (32.56–69.12)
COM (GB)	97.56 (83.92–99.65)	48.91 (29.74–65.58)

SVF (RSF)	92.02 (69.39–98.12)	58.29 (38.71–73.57)
SVF (EST)	95.12 (81.87–98.75)	49.49 (30.17–66.17)
SVF (GB)	97.05 (80.90–99.58)	51.51 (31.40–68.39)

DEA (RSF)	86.71 (62.54–95.76)	63.73 (44.32–77.92)
DEA (EST)	87.53 (64.60–96.02)	64.59 (45.35–78.54)
DEA (GB)	87.37 (64.14–95.95)	59.89 (40.10–75.01)

IMG (RSF)	86.33 (70.10–94.10)	66.89 (41.08–83.37)
IMG (EST)	85.55 (68.49–93.76)	65.47 (44.29–80.23)
IMG (GB)	91.30 (75.25–97.13)	67.97 (46.96–82.10)

PPE (RSF)	78.95 (60.49–89.48)	60.12 (38.69–76.13)
PPE (EST)	89.27 (73.75–95.86)	60.52 (37.19–77.50)
PPE (GB)	94.34 (79.20–98.55)	60.59 (36.39–78.01)

**Fig. 2:**
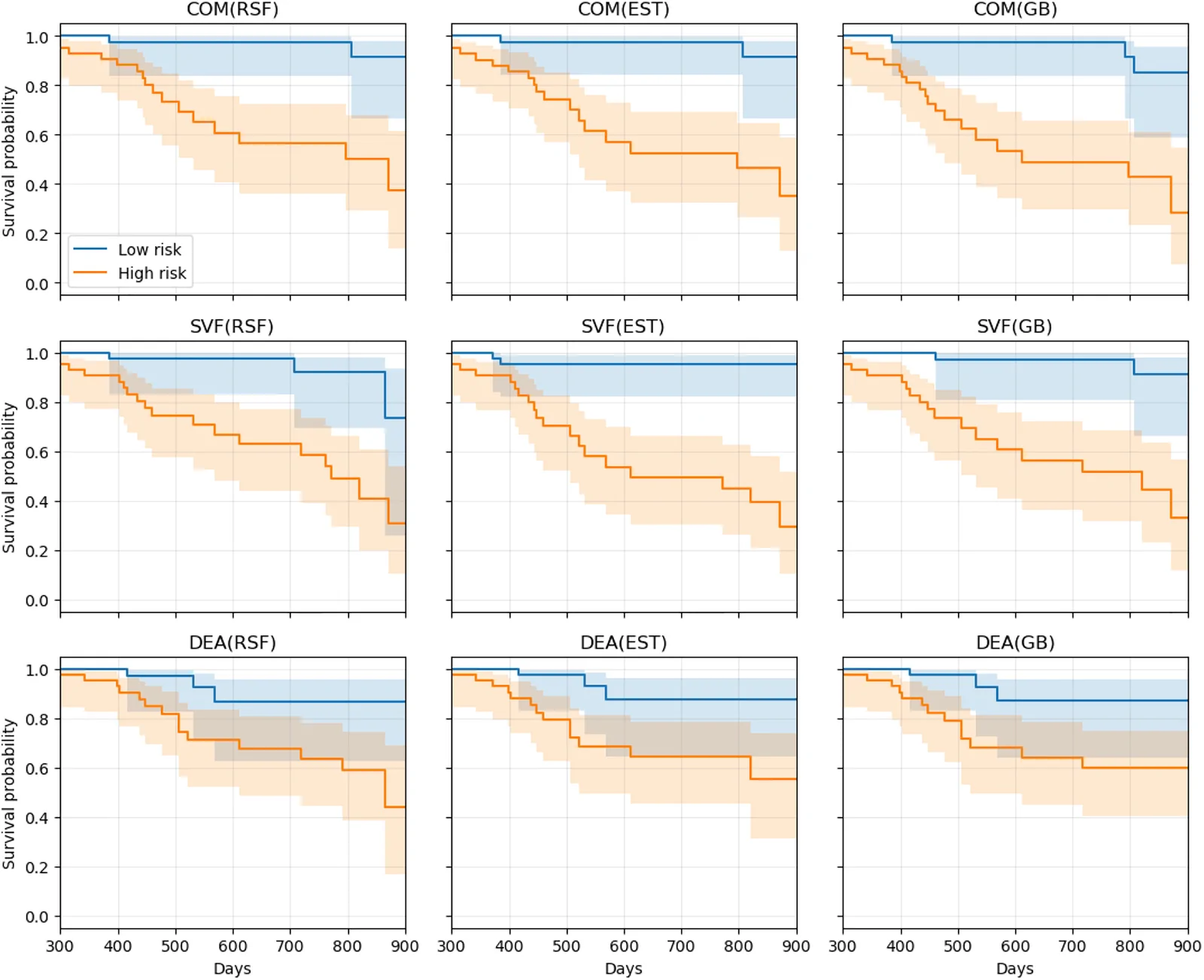
K–M survival curves comparing predicted low- and high-risk groups for the COM, SVF and DEA categories.

**Table 3:** Comparison of mean values of selected SVF features between low-risk and high-risk groups across predictive models.

Variable	Model	Mean (SD)		p-value
		Low Risk	High Risk	
	RSF	19.55 (4.0)	8.58 (3.68)	**<0.001**
Unique animals count	EST	19.23 (3.85)	8.81 (3.14)	**<0.001**
	GB	18.41 (4.58)	8.34 (3.03)	**<0.001**

Log mean	RSF	3.82 (0.24)	4.59 (0.34)	**<0.001**
language-use	EST	3.77 (0.20)	4.65 (0.30)	**<0.001**
frequency of words	GB	3.79 (0.16)	4.64 (0.32)	**<0.001**

Standard deviation of	RSF	9.24 (0.91)	10.79 (2.20)	**<0.001**
F1 bandwidth	EST	9.40 (1.05)	10.78 (1.94)	**<0.001**
falling slope	GB	9.13 (0.90)	10.61 (2.08)	**<0.001**

Proportion of	RSF	-	-	-
non-animal words	EST	0.11 (0.14)	0.63 (0.16)	**<0.001**
excluding functional	GB	0.11 (0.13)	0.61 (0.18)	**<0.001**

Mean of	RSF	-	-	-
F0 bandwidth	EST	0.40 (0.09)	0.55 (0.10)	**<0.001**
rising slope	GB	0.38 (0.08)	0.54 (0.11)	**<0.001**

Percentile 20–80	RSF	0.026 (0.004)	0.024 (0.005)	0.058
range of 500–1500	EST	-	-	-
Hz spectral slope	GB	-	-	-

Mean of	RSF	0.223 (0.035)	0.247 (0.037)	**0.002**
harmonic-to-noise	EST	-	-	-
ratio rising slope	GB	-	-	

Mean language-use	RSF	-	-	-
frequency of words	EST	.5· 10^–4^ (.9· 10^–4^)	4.4· 10^–4^ (2.4· 10^–4^)	**<0.001**
excluding functional	GB	-	-	-

Skew of	RSF	-	-	-
F1 bandwidth	EST	−0.083 (0.478)	−0.327 (0.600)	**0.040**
	GB	-	-	-

Ratio of	RSF	-	-	-
unique to total	EST	0.96 (0.03)	0.82 (0.15)	**<0.001**
animal responses	GB	-	-	-

## 4 Discussion

The primary aim of this study was to evaluate the prognostic value of spontaneous speech features within an established longitudinal survival-analysis framework and to compare their performance for predicting MCI-to-dementia conversion with established cognitive composite measures.

These results contribute to the growing literature on digital biomarkers by addressing an important gap. Many prior studies using speech or other digital signals have predominantly employed cross-sectional designs or focused on diagnostic classification rather than longitudinal risk estimation [[36], [37], [38]]. While a limited number of recent studies have begun to explore longitudinal prediction using speech-derived features, including cohort analyzes leveraging language models (e.g. [39]), the field remains largely dominated by cross-sectional diagnostic classification approaches, and prospective time-to-event modeling of clinical conversion remains relatively scarce [40]. By embedding speech features within a survival modeling framework and benchmarking them against composite cognitive measures, the present study provides evidence that speech-based markers retain prognostic validity when evaluated against a clinically meaningful longitudinal endpoint. This approach strengthens the clinical interpretability of speech-derived digital markers beyond diagnostic classification paradigms. Beyond overall model performance, task-level analyzes revealed marked heterogeneity in the prognostic value of different speech elicitation paradigms. Features derived from the SVF task consistently yielded the strongest discriminative performance. In contrast, IMG and PPE showed weaker discriminative ability, performing worse than DEA.

Models incorporating features from all speech tasks were largely driven by the SFV category, as the feature-selection algorithm retained only variables derived from this task. Therefore, the apparent advantage of the combined-task models should not be interpreted as evidence of complementary prognostic information across speech elicitation paradigms. Instead, these results suggest that SVF-derived features carried the most relevant predictive signal for survival risk stratification, while features from the other tasks contributed limited additional value.

The prominent contribution of SVF aligns with prior work from our group [7], in which SVF-based speech features emerged as robust predictors of clinically relevant outcomes. The present findings extend this evidence by demonstrating that SVF retains strong prognostic utility within a survival modeling framework, even when features from other speech elicitation tasks are made available to the model.

SVF-derived speech predictors showed good prognostic performance for MCI-to-dementia conversion, although they did not reach the discriminative accuracy observed for cognitive composites. Nevertheless, the performance achieved by SVF is notable given the brevity and simplicity of the task. Unlike composites, SVF can be obtained from a short speech sample and processed automatically, making it potentially suitable for repeated assessments and remote monitoring. Thus, the clinical value of SVF may lie less in replacing cognitive composites and more in extending prognostic assessment to settings where comprehensive neuropsychological testing is impractical.

Some limitations should be considered when interpreting these results. First, follow-up duration was relatively limited, and there was a substantial proportion of censored observations. Because participants were recruited over an extended period, those enrolled later necessarily contributed shorter observation windows. Consequently, some individuals classified as stable MCI had approximately one year of follow-up and may still convert to dementia with longer observation.

Short follow-up increases uncertainty around censored observations, limits the precision of longer-term risk estimates, and may reduce the ability to capture late MCI-to-dementia converters. Therefore, the present findings should be interpreted primarily as evidence of short-to medium-term prognostic discrimination within the available follow-up period. Although this time horizon was sufficient to support the survival analyzes conducted in this study, longer follow-up would allow more precise estimation of late-occurring events, reduce uncertainty associated with censoring, and potentially improve both model discrimination and calibration.

Second, the study was conducted in a single center, and the findings have not yet been externally replicated. This is particularly relevant for speech-based biomarkers, as they may be influenced by language, dialectal variation, or sociolinguistic factors. Therefore, although the present findings support the prognostic potential of speech-derived features within this cohort, their generalization to other clinical settings, populations, and speech- acquisition protocols remain uncertain. Future studies should externally validate these models in independent and multilingual cohorts.

In conclusion, SVF-derived speech features demonstrated good prognostic performance for MCI-to-dementia conversion, although cognitive composites remained the strongest predictors. These findings support the potential utility of SVF as a scalable, low-burden complement to standard cognitive assessment rather than as a replacement. Future research should evaluate the incremental value of speech-based markers beyond standard clinical assessments, examine their integration into routine clinical workflows, and determine their robustness in cohorts with longer and more homogeneous follow-up and more diverse populations.

## Ethical considerations

This study was approved by the ethics committee of the Hospital Universitari de Bellvitge (Barcelona) (ref. PR007/22) in compliance with Spanish biomedical laws (Law 14/2007, July 3, regarding biomedical research; Royal Decree 1716/2011, November 18) on 10 March 2022 and adhered to the principles of the Declaration of Helsinki.

## Consent to participate

All participants were provided with detailed information about the study’s objectives, procedures, and potential risks before signing an informed consent form for the administration of the speech protocol.

## Generative AI declaration

During the preparation of this manuscript, the authors used ChatGPT for the purpose of proofreading, which represented a small proportion of the writing process. The authors subsequently reviewed and edited the content as necessary and assume full responsibility for the accuracy and integrity of the published work.

## Funding

This project has received funding from R&D Missions in the Artificial Intelligence program, which is part of the Spain Digital 2025 Agenda and the National Artificial Intelligence Strategy and financed by the European Union through Next Generation EU funds (project TARTAGLIA, exp. MIA.2021.M02.0005).

This project has also received funding from the Instituto de Salud Carlos III (ISCIII) Acción Estratégica en Salud, integrated in the Spanish National RCDCI Plan and financed by ISCIII Subdirección General de Evaluación and the Fondo Europeo de Desarrollo Re- gional (FEDER-Una manera de hacer Europa) grant PI19/00335 and PI25/00067 awarded to M.M., grant PI17/01474 awarded to M.B., grants AC17/00100, PI19/01301 and PI22/01403 awarded to A.R. and by the European Union Joint program-Neurodegenerative Disease Research (JPND) Multinational research projects on Personalized Medicine for Neurodegenerative Diseases/Instituto de Salud Carlos III grant AC19/00097 awarded to A.R. and grant FI24/00029 from the Instituto de Salud Carlos III (ISCIII) awarded to C.O. M.M. received funding support from the European Union’s Horizon 2020 research and innovation program under the Marie Sklodowska-Curie grant agreement N° 796706. A.R. and M.B. received support from the European Union/EFPIA Innovative Medicines Initiative Joint undertaking ADAPTED and MOPEAD projects (grant numbers 115975 and 115985, respectively). P.G.G. is supported by CIBERNED employment plan (CNV-304-PRF-866).

A.C. received support from the Instituto de Salud Carlos III (ISCIII) under the grant Sara Borrell (CD22/00125) and the Spanish Ministry of Science and Innovation, Proyectos de Generación de Conocimiento grant PID2021–122473OA-I00.

## CRediT authorship contribution statement

**Adrián Hinojosa-Calleja:** Writing – review & editing, Writing – original draft, Software, Methodology, Formal analysis, Data curation. **Berta Calm:** Writing – review & editing, Writing – original draft, Software, Methodology, Formal analysis, Data curation. **Montserrat Alegret:** Writing – review & editing, Investigation. **Ana Espinosa:** Writing – review & editing, Investigation. **Amanda Cano:** Writing – review & editing, Investigation. **Maria Victoria Fernández:** Writing – review & editing, Investigation. **Andrea Miguel:** Writing – review & editing, Investigation. **Ariadna Solivar:** Writing – original draft, Investigation. **Alejandro Valenzuela-Seba:** Writing – review & editing, Investigation. **Pablo García-González:** Writing – review & editing, Investigation. **Raquel Puerta:** Writing – review & editing, Investigation. **Clàudia Olivé:** Writing – review & editing, Investigation. **Maria Capdevila-Bayo:** Writing – review & editing, Investigation. **Álvaro Muñoz-Morales:** Writing – review & editing, Investigation. **Paula Bayón-Buján:** Writing – review & editing, Investigation. **Marta Rovira-Bruna:** Writing – review & editing, Investigation. **Maider Iza:** Writing – review & editing, Investigation. **Laura Montreal:** Writing – review & editing, Investigation. **Maitee Rosende-Roca:** Writing – review & editing, Investigation. **Pilar Sanz-Cartagena:** Writing – review & editing, Investigation. **Yahveth Cantero-Fortiz:** Writing – review & editing. **Miren Jone Gurruchaga:** Writing – review & editing, Conceptualization. **Lluis Tarraga:** Writing – review & editing. **Xavier Montalbán:** Writing – review & editing. **Mercè Boada:** Writing – review & editing, Conceptualization. **Agustín Ruiz:** Writing – review & editing, Project administration, Conceptualization. **Marta Marquié:** Writing – review & editing, Project administration, Investigation, Conceptualization. **Sergi Valero:** Writing – review & editing, Project administration, Conceptualization.

## Declaration of competing interest

The authors declare the following financial interests/personal relationships which may be considered as potential competing interests:

Agustin Ruiz reports a relationship with Landsteiner Genmed that includes: board membership and equity or stocks. Agustin Ruiz reports a relationship with Grifols SA that includes: board membership. Merce Boada reports a relationship with Araclon Biotech S.L. that includes: consulting or advisory, funding grants, and speaking and lecture fees. Merce Boada reports a relationship with Avid that includes: consulting or advisory. Merce Boada reports a relationship with Grifols SA that includes: consulting or advisory, funding grants, and speaking and lecture fees. Merce Boada reports a relationship with Lilly SA that includes: consulting or advisory and funding grants. Merce Boada reports a relationship with Nutricia SRL that includes: consulting or advisory, funding grants, and speaking and lecture fees. Merce Boada reports a relationship with Roche Pharma SA that includes: consulting or advisory, funding grants, and speaking and lecture fees. Merce Boada reports a relationship with Eisai that includes: consulting or advisory. Merce Boada reports a relationship with Laboratorios Servier SL that includes: funding grants and speaking and lecture fees. Merce Boada reports a relationship with Biogen Research Limited that includes: funding grants and speaking and lecture fees. Merce Boada reports a relationship with AbbVie that includes: funding grants. Merce Boada reports a relationship with Bioiberica that includes: funding grants. Merce Boada reports a relationship with Merck Sharp & Dohme that includes: funding grants. Merce Boada reports a relationship with Kyowa Hakko Kirin that includes: funding grants. Merce Boada reports a relationship with Oryzon Genomics, S.A. that includes: funding grants. Merce Boada reports a relationship with Piramal Imaging Limited that includes: funding grants. Merce Boada reports a relationship with Schwabe Pharma Iberica that includes: funding grants. Marta Marquie reports a relationship with F. Hoffmann-La Roche Ltd that includes: consulting or advisory. Marta Marquie reports a relationship with Araclon that includes: board membership. If there are other authors, they declare that they have no known competing financial interests or personal relationships that could have appeared to influence the work reported in this paper.

## Data Availability

The datasets generated and/or analyzed during the current study are not publicly available because they contain privacy-sensitive human data, but they are available from the corresponding author on reasonable request.

## References

[1] Grand J.H., Caspar S., MacDonald S.W. (2011). Clinical features and multidisciplinary approaches to dementia care. J Multidiscip Healthc.

[2] World Health Organization (2021). https://apps.who.int/iris/bitstream/handle/10665/344707/9789240034624-eng.pdf.

[3] World Health Organization (2017). https://iris.who.int/server/api/core/bitstreams/2098e5be-c7f3-4365-aebf-36204459794f/content.

[4] Martínez-Nicolás I., Llorente T.E., Martínez-Sánchez F., Meilán J.J.G. (2021). Ten years of research on automatic voice and speech analysis of people with Alzheimer's disease and mild cognitive impairment: a systematic review article. Front Psychol.

[5] García-Gutiérrez F., Marquié M., Muñoz N. (2023). Harnessing acoustic speech parameters to decipher amyloid status in individuals with mild cognitive impairment. Front Neurosci.

[6] García-Gutiérrez F., Alegret M., Marquié M. (2024). Unveiling the sound of the cognitive status: machine learning-based speech analysis in the Alzheimer's disease spectrum. Alzheimers Res Ther.

[7] Blazquez-Folch J., Calm B., Hinojosa-Calleja A. (2026). Listening for Alzheimer’s clues: machine learning analysis of multidomain speech features for cognitive impairment screening. Front Aging Neurosci.

[8] Sperling R.A., Karlawish J., Johnson K.A. (2013). Preclinical Alzheimer disease—the challenges ahead. Nat Rev Neurol.

[9] Szatloczki G., Hoffmann I., Vincze V., Kalman J., Pakaski M. (2015). Speaking in Alzheimer’s disease, is that an early sign? Importance of changes in language abilities in Alzheimer’s disease. Front Aging Neurosci.

[10] Tóth L., Hoffmann I., Gosztolya G. (2018). A speech recognition-based solution for the automatic detection of mild cognitive impairment from spontaneous speech. Curr Alzheimer Res.

[11] Tröger J., Baykara E., Zhao J. (2022). Validation of the remote automated ki: e speech biomarker for cognition in mild cognitive impairment: verification and validation following dime v3 framework. Digi Biomark.

[12] Nakagawa T., Ishida M., Naito J. (2020). Prediction of conversion to Alzheimer’s dis- ease using deep survival analysis of mri images. Brain Commun.

[13] Song S., Asken B., Armstrong M.J., Yang Y., Li Z., Initiative A.D.N. (2023). Predicting progression to clinical Alzheimer’s disease dementia using the random survival forest. J Alzheimers Dis.

[14] Pölsterl S., Sarasua I., Gutiérrez-Becker B., Wachinger C. (2019). Joint European Conference on Machine Learning and Knowledge Discovery in Databases.

[15] Orozco-Sanchez J., Tamez-Peña J. (2020). Prediction of MCI to AD risk of conversion survival models: qMRI vs CSF measures and cognitive assessments. Med Imaging 2020 Comput -Aided Diagn.

[16] Spooner A., Chen E., Sowmya A. (2020). A comparison of machine learning methods for survival analysis of high-dimensional clinical data for dementia prediction. Sci Rep.

[17] Mirabnahrazam G., Ma D., Beaulac C. (2023). Predicting time-to-conversion for dementia of Alzheimer’s type using multi-modal deep survival analysis. Neurobiol Aging.

[18] Musto H., Stamate D., Pu I., Stahl D. (2023). International Conference on Computational Collective Intelligence.

[19] Sarica A., Aracri F., Bianco M.G. (2023). Explainability of random survival forests in predicting conversion risk from mild cognitive impairment to Alzheimer’s disease. Brain Inform.

[20] Sarica A., Pelagi A., Aracri F. (2024). Sex Differences in Conversion Risk from Mild Cognitive Impairment to Alzheimer’s Disease: An Explainable Machine Learning Study with Random Survival Forests and SHAP. Brain Sci.

[21] Guan T., Shang L., Yang P. (2025). Joint ensemble learning-based risk prediction of Alzheimer’s disease among mild cognitive impairment patients. J Prev Alzheimers Dis.

[22] Jahani S., Roshanaei G., Tapak L., Initiative A.D.N. (2025). Assessing the accuracy of survival machine learning and traditional statistical models for Alzheimer's disease prediction over time: a study on the adni cohort. BMC Med Res Methodol.

[23] Boada M., Tárraga L., Hernández I. (2014). Design of a comprehensive Alzheimer's disease clinic and research center in Spain to meet critical patient and family needs. Alzheimers Dement.

[24] Garcia-González P., Puerta R., Muñoz-Morales A. (2025). Long-read sequencing reveals two common intragenic haplotypes within each of the APOE ε3 and ε4 alleles in the Spanish population Corresponding. medRxiv.

[25] Jessen F., Amariglio R.E., Van Boxtel M. (2014). A conceptual framework for research on subjective cognitive decline in preclinical Alzheimer's disease. Alzheimers Dementi.

[26] Alegret M., Espinosa A., Vinyes-Junqué G. (2012). Normative data of a brief neuropsychological battery for Spanish individuals older than 49. J Clin Exp Neuropsychol.

[27] Cummings L. (2019). Describing the cookie theft picture: sources of breakdown in Alzheimer's dementia. Pragmat Soc.

[28] Radford A., Kim J.W., Xu T., Brockman G., McLeavey C., Sutskever I. (2023). International Conference on Machine Learning.

[29] Qi P., Zhang Y., Zhang Y., Bolton J., Manning C.D. (2020). Proceedings of the 58th Annual Meeting of the Association for Computational Linguistics: System Demonstrations.

[30] Stekhoven D.J., Bühlmann P. (2012). Missforest—non-parametric missing value imputation for mixed-type data. Bioinformatics.

[31] Wang P., Li Y., Reddy C.K. (2019). Machine learning for survival analysis: a survey. ACM Comput Surv (CSUR).

[32] Qi S-A, Kumar N., Farrokh M. (2023). An effective meaningful way to evaluate survival models. Proc Mach Learn Res.

[33] Lambert J., Chevret S. (2016). Summary measure of discrimination in survival models based on cumulative/dynamic time-dependent roc curves. Stat Methods Med Res.

[34] Pölsterl S. (2020). scikit-survival: a library for time-to-event analysis built on top of scikit- learn. J Mach Learn Res.

[35] Zhou H., Xin Y., Li S. (2023). A diabetes prediction model based on boruta feature selection and ensemble learning. BMC Bioinformatics.

[36] König A., Satt A., Sorin A. (2015). Automatic speech analysis for the assessment of patients with predementia and Alzheimer's disease. Alzheimers Dement.

[37] Fraser K.C., Meltzer J.A., Rudzicz F. (2015). Linguistic features identify Alzheimer's disease in narrative speech. J Alzheimers Dis.

[38] Luz S, Haider F, de la Fuente Garcia S, Fromm D, and MacWhinney B, “Alzheimer’s dementia recognition through spontaneous speech,” (2021), 10.3389/fcomp.2021.780169. 10.3389/fcomp.2021.780169 PMC8920352 35291512

[39] Amini S., Hao B., Yang J. (2024). Prediction of Alzheimer's disease progression within 6 years using speech: a novel approach leveraging language models. Alzheimers Dement.

[40] Mueller K.D., Koscik R.L., Hermann B.P., Johnson S.C., Turkstra L.S. (2018). Declines in connected language are associated with very early mild cognitive impairment: results from the Wisconsin registry for Alzheimer's prevention. Front Aging Neurosci.

