# Supplementary material for: Survival analysis of spontaneous speech for predicting mild cognitive impairment-to-dementia conversion
Source: J Prev Alzheimers Dis. 2026 Nov 3;13(10):100692. doi: 10.1016/j.tjpad.2026.100692 (PMC13756245; doi:10.1016/j.tjpad.2026.100692)
Supplement: Supplementary file 1 [file mmc1.pdf]

Supplementary information

Ethical considerations

This study was approved by the ethics committee of the Hospital Universitari de Bellvitge (Barcelona) (ref. PR007/22) in compliance with Spanish biomedical laws (Law 14/2007, July 3, regarding biomedical research; Royal Decree 1716/2011, November 18) on 10 March 2022 and adhered to the principles of the Declaration of Helsinki.

Consent to participate

All participants were provided with detailed information about the study's objectives, procedures, and potential risks before signing an informed consent form for the administration of the speech protocol.

Author contributions

A.H.C.: Data curation, formal analysis, software, methodology, writing – original draft, writing – review & editing; B.C.: Data curation, formal analysis, software, methodology, writing – original draft, writing – review & editing; M.A.: Investigation, writing – review & editing; A.E.: Investigation, writing – review & editing; A.C.: Investigation, writing – review & editing; M.V.F.: Investigation, writing – review & editing; A.M.: Investigation, writing – review & editing; A.S.: Investigation, writing – review & editing; A.V.S.: Investigation, writing – review & editing; P.G.G.: Investigation, writing – review & editing; R.P.: Investigation, writing – review & editing; C.O.: Investigation, writing – review & editing; M.C.B.: Investigation, writing – review & editing; A.M.M.: Investigation, writing – review & editing; P.B.B.: Investigation, writing – review & editing; MRB.: Investigation, writing – review & editing; M.I.: Investigation, writing – review & editing; L.M.: Investigation, writing

– review & editing; M.R.R.: Investigation, writing – review & editing; P.S.C.: Investigation, writing – review & editing; Y.C.F.: Writing – review & editing; M.J.G.: Conceptualization, writing – review & editing; L.T.: Writing – review & editing; X.M.: Writing – review & edit- ing; M.B.: Conceptualization, writing – review & editing; A.R.: Conceptualization, project administration, writing – review & editing; M.M.: Conceptualization, Investigation, project administration, writing – review & editing; S.V.: Conceptualization, project administration, writing – review & editing.

Availability of data and materials

The datasets generated and/or analyzed during the current study are not publicly available because they contain privacy-sensitive human data, but they are available from the corresponding author on reasonable request.

Competing interests

A.R. is a member of the scientific advisory board of Landsteiner Genmed and Grifols SA. He has stocks of Landsteiner Genmed. M.B. has consulted for Araclon, Avid, Grifols, Lilly, Nutricia, Roche, Eisai and Servier. She received fees from lectures and funds for research from Araclon, Biogen, Grifols, Nutricia, Roche and Servier. She reports grants/research funding from Abbvie, Araclon, Biogen Research Limited, Bioiberica, Grifols, Lilly, S.A, Merck Sharp & Dohme, Kyowa Hakko Kirin, Laboratorios Servier, Nutricia SRL, Oryzon Genomics, Piramal Imaging Limited, Roche Pharma SA, and Schwabe Farma Iberica SLU, all outside the submitted work. She has not received personal compensation from these organizations. M.M. has consulted for F. Hoffmann-La Roche Ltd and is a member of the Scientific Advisory Board of Biomarkers of Araclon. The rest of the authors declare that they have no competing interests.

Generative AI Declaration

During the preparation of this manuscript, the authors used ChatGPT for the purpose of proofreading, which represented a small proportion of the writing process. The authors subsequently reviewed and edited the content as necessary and assume full responsibility for the accuracy and integrity of the published work.
